# Aurivillius’s “Neue oder wenig bekannte ColeopteraLongicornia” (1886–1927), the correct years and page numbers

**DOI:** 10.3897/zookeys.911.48684

**Published:** 2020-02-12

**Authors:** Mei-Ying Lin, Si-Qin Ge

**Affiliations:** 1 Key Laboratory of Zoological Systematics and Evolution, Institute of Zoology, Chinese Academy of Sciences, 1–5 Beichen West Road, Chaoyang Dist., Beijing, 100101, China Institute of Zoology, Chinese Academy of Sciences Beijing China

**Keywords:** Correction, misunderstanding, publication date, reference, years of submission, Zoological Record

## Abstract

Aurivillius’s work entitled “Neue oder wenig bekannte ColeopteraLongicornia” was published in parts over a period of over four decades. There were two page numbers on most pages of these publications, one ordered by Aurivillius, the other by the journal. Historically, different authors have used different page numbers, and sometimes different years for these publications, which has caused chaos in the citations. Herein, accurate dates of publications for this work, and correct page numbers that should be used are provided and discussed.

## Introduction

Christopher Aurivillius (1853–1928) was a very important Swedish entomologist, who published 67 references regarding Cerambycidae from 1886 to 1929 (Tavakilian and Chevillotte 2019). Among them, 20 parts were titled as “Neue oder wenig bekannte ColeopteraLongicornia” and numbered from 4 to 23. Most of them (except the 8^th^ part) have two page numbers printed on each page, both of which have been cited by many different authors. In order to determine the correct page numbers and the accurate dates of publication for this significant work, we analyzed all the Cerambycidae literature of Aurivillius.

## Materials and methods

### Methods of literature collecting

We accessed literature in three ways for this study: a) downloaded pdf files from the Biodiversity Heritage Library: http://www.biodiversitylibrary.org/; b) copied the original pages directly from library holdings (the first author visited the libraries of the Institute of Zoology, Chinese Academy of Sciences, Beijing, China; National Science Library, Chinese Academy of Science, Beijing, China; Muséum national d’Histoire naturelle, Paris, France; Division of Plant Industry, Florida State Collection of Arthropods, Gainesville, Florida, USA; and the National Museum of Natural History (Smithsonian Institution), Washington DC, USA, etc.); c) solicited help from colleagues (especially G. Tavakilian and S. Lingafelter).

### Dating the publications

In researching the dates of publication for this work, we consulted five points of reference: a) date printed on first and last pages; b) date shown by the Zoological Record; c) date used by Aurivillius’s catalogues; d) date used by literature citing related references; e) date printed on original wrapper.

## Results

### Historically different ways of citing page numbers

We examined most of the literature citing Aurivillius’s “Neue oder wenig bekannte ColeopteraLongicornia” published in the journal “Arkiv för zoologi”, and gathered the results herein.

a) Citation using the journal’s page numbers: Aurivillius 1912, 1922, 1923, 1928; Breuning 1939, 1940, 1944, 1950, 1956, 1958–1969; Lane 1950; Quentin 1956; Podaný 1968, 1971; Gressitt and Rondon 1970; Rondon and Breuning 1970; Hüdepohl 1985, 1988, 1989, 1990; Lee 1987; Niisato 1989, 2007; Nakamura et al. 1992; Napp 1993; Nýlander 1998; Makihara 1999; Heffern 2002; Makihara and Woro 2002; Morati and Huet 2004; Ohbayashi and Niisato 2007; Bousquet et al. 2009; Morati and Bentanachs 2009; Bentanachs et al. 2010; Sudre et al. 2010; Juhel 2011; Jiroux 2011; Vitali 2011; Weigel and Skale 2011; Vitali and Vitali 2011; Wallin et al. 2014; Lin 2015; Vitali et al. 2017.

b) Citation using both Aurivillius’s and the journal’s page numbers, but considering journal numbers as more important: Löbl and Smetana 2010; Heffern 2011; Viktora 2013, 2015a, 2019; Viktora and Tichý 2017; Lin and Yang 2019.

c) Citations using both Aurivillius’s and the journal’s page numbers, but considering Aurivillius’s numbers as more important: Hüdepohl 1992; Juhel and Bentanachs 2010.

d) Citations using Aurivillius’s page numbers: Tavakilian 1991; Martins and Galileo 1992; Martins 1997, 1998, 1999, 2002, 2005, 2007, 2009, 2011, 2014; Adlbauer 1998; 2002a, 2002b, 2013; Napp and Mermudes 1999; Vives and Heffern 2001; Vives and Abang 2003; Hüdepohl and Heffern 2004; Heffern 2005; Monné 2005a, 2005b, 2012, 2019a, 2019b, 2019c, 2019d; Monné and Napp 2005; McCarty 2006; Napp 2007; Yokio and Niisato 2009; Juhel and Bentanachs 2009a, 2011, 2012; Vives 2009, 2012, 2015a, 2015b, 2015c, 2015d, 2017; Martins and Santos-Silva 2010; Lin and Yang 2011; Juhel 2012, 2014a, 2015; Monné et al. 2012, 2016; Huang et al. 2014; Jiroux et al. 2014; Lingafelter et al. 2014; Miroshnikov 2014; Gouverneur 2015; Monné and Monné 2015, 2017; Viktora 2015b; Rousset et al. 2016; Santos-Silva and Galileo 2016; Sudre et al. 2016; Viktora and Tichý 2016; Vitali 2016, 2018; Yan and Chen 2016; Yokio and Heffern 2016; Bentanachs and Jiroux 2017; Santos-Silva and Botero 2017. Note that some of these citations could have followed the Titan database (Tavakilian and Chevillotte (2019) since they also cited that database (e.g., Huang et al. 2014; Gouverneur 2015).

e) Random citation method: Sometimes using the journal’s page numbers and sometimes using Aurivillius’s page numbers in the same paper: Gressitt 1940, 1951; Lingafelter and Hoebeke 2002; Juhel and Bentanachs 2009b; Ślipiński and Escalona 2013; Juhel 2014b, 2016; Nakamura et al. 2014; Lin 2017; Bezark 2019; Lazarev and Murzin 2019.

### Common errors encountered when citing this series of papers

The errors occurred in the date (see Table 1), page numbers (see Table 1), information regarding the figures and plates, part numbers, first and last page numbers, journal volume numbers, and so on.

**Figure 1.**
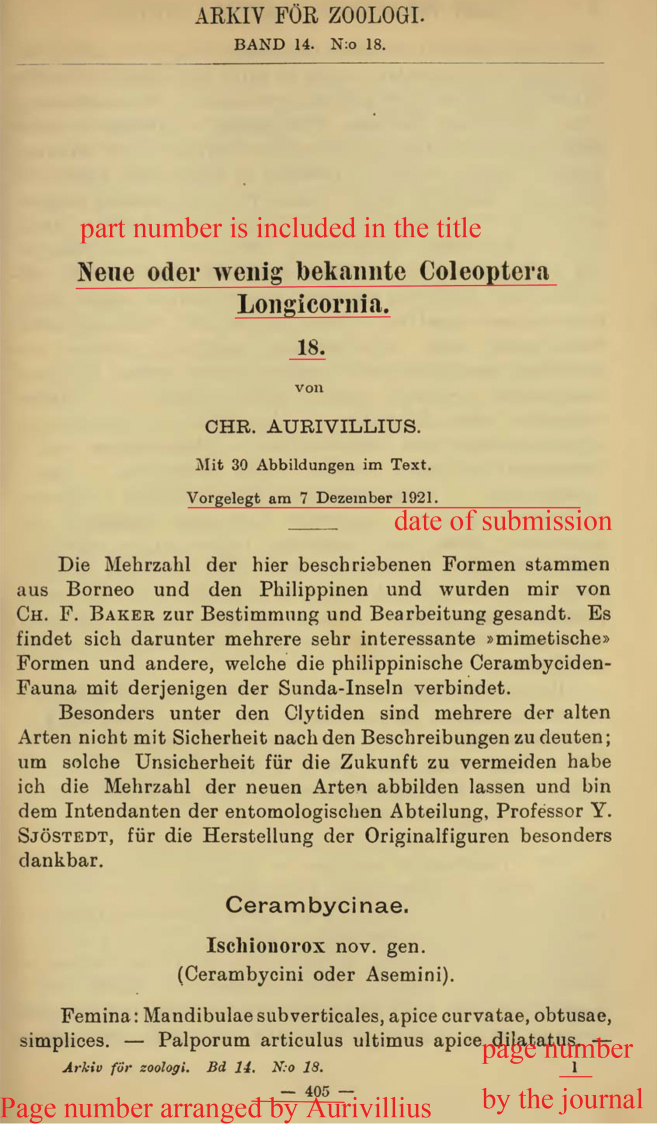
First page of part 18, showing the title, date of submission, journal’s page number and Aurivillius’s page number.

a) Errors regarding the separate plates. There were two kinds of figures in this work, text-figures were inside the content and provided with continuous numbers (see Table 1), while end-plates were printed as separate plates, normally numbered from one. The former can be ignored in the reference, while the latter should be added. For example, Hüdepohl and Heffern (2004) wrote the reference as “Aurivillius, C. 1907. Neue oder wenig bekannte ColeopteraLongicornia. 9. Arkiv för zoologi. 3(18): 93–131. 9 fig.”, the “9 fig.” would be better stated as “pl. 1: fig. 1–9” or “1 pl.”, since there were seven text-figures (figs 35–41) inside the content too, which might cause misunderstanding. Sometimes the separate end-plate was missing in the reference (Monné 2005a, 2005b; Vives 2009; Lingafelter et al. 2014; Monné 2019a, 2019b, 2019d; Monné et al. 2016, missing the plate when citing part 16; Monné 2019a, 2019b, 2019d missing the plate when citing part 22).

**Table 1.** Bibliographic details of the series “Neue oder wenig bekannte ColeopteraLongicornia” by Aurivillius (1886–1927). AFZ: Arkiv för zoologi; ET: Entomologisk Tidskrift; ICZN: International Code for Zoological Nomenclature.

Publishing year	Title	Journal name	Volume: page numbers by the journal	Page numbers arranged by Aurivillius	Species numbers	Text-figure numbers (inside content)	End-plates (separated from content)	Date of submission (on first page)	Released / printed date (on last page)	Came out date (for the volume normally)
1886	Nya ColeopteraLongicornia	ET	7(2): 89–94.	none (1–6 concluded herein)	1–5	none	none			12 July 1886
1887	Nya ColeopteraLongicornia. II.	ET	8(4): 191–197.	none (7–14 concluded herein)	6–10	figs 1–3.	none			31 Dec. 1887
1891	Neue ColeopteraLongicornia. III	ET	12 (2): 97–106.	15–24	11–20	figs 1–6.	none			18 June 1891
Aurivillius began to arrange continuous page numbers for this series as a whole from the third part in 1891. There were 14 pages for the previous two parts titled “Nya ColeopteraLongicornia”, so the third part started on page 15. Though the third part did not use the exact same title, the meaning of the titles were the same.										
1893	Neue oder wenig bekannte ColeopteraLongicornia. 4.	ET	14(3): 177–186.	25–34	21–30	figs 1–12.	none			25 May 1893
Aurivillius fixed the title as “Neue oder wenig bekannte ColeopteraLongicornia” from part 4 in 1893, then kept it to the end. In this table, parts 5 to 23 did not repeat the title but only marked the part numbers. Species number 21 was related to one new genus whose type species was not described by Aurivillius. Later, genera were not numbered again.										
1897	5.	ET	18(4): 241–248.	35–42	31–41	none	Pl. 3: figs 1–8.			19 Jan. 1898
According to Derksen and Scheiding (1963), the date of publication is 1897. All references we searched and the Titan database (Tavakilian and Chevillotte 2019) used 1897, also the Zoological Record indicated 1897. However, the website https://www.biodiversitylibrary.org/item/89782#page/8/mode/1up indicated that the publication date 19 Jan. 1898. “Utgifvet den 19 januari 1898” (= issued/published on January 19th 1898) (printed low on the back side of the original wrapper) should be the official publication date (personal communication with Mikael Sörensson on 10 December 2019).										
1899	6.	ET	20(4): 259–265.	51–57	42–52	figs 13–17.	none			23 Jan. 1900
For Aurivillius’s own page numbers, the fifth part ended with page 42, while the sixth part began with page 51. That was because Aurivillius gave one page for each figure, therefore eight figures took the pages 43 to 50. The figures inside the sixth part continued the numbers from the fourth part, while the figures in fifth part arranged as a separate plate were not numbered continuously. “Utgifvet den 23 januari 1900” (= issued/published on January 23rd 1900) (printed low on the back side of the original wrapper) should be the official publication date (personal communication with Mikael Sörensson on 10 December 2019). Also the website https://www.biodiversitylibrary.org/item/43633#page/584/mode/1up indicated the publication date as 23 Jan. 1900. We chose 1899 based on the Zoological Record and Derksen and Scheiding (1963).										
1902	7.	ET	23: 207–224.	59–76 (printed as:1, 2, 61–76)	53–75	figs 18–26.	none			2 Sept. 1902
Aurivillius’s page number did not have page 58. That was because every new part began with odd numbers. Therefore, when the previous part ended with odd numbers (parts 6, 9, 12, 13, 15, 16, 17, 19, 20, 22), one even number would be taken by an empty page (page numbers 58, 228, 264, 334, 404, 480, 502) or by a separated plate (page numbers 132, 360, 548).										
1903	8.	AFZ	1: 313–328.	Not printed (but should be 77–92 in conclusion)	76–96	figs 27–34.	none	14 Oct. 1903	27 Nov. 1903	21 Jan. 1904
Aurivillius chose the journal AFZ since the eighth part and did not change it again. In this first printing in the AFZ journal, the journal did not include Aurivillius’s own page numbers. But from the ninth part, the AFZ printed both the journal’s page number on top and Aurivillius’ own page number at bottom. The date of publication of the eighth part is confusing. The printing date on the last page is “Tryckt den 27 november 1903”, so it should be 1903. However, Aurivillius (1912; 1922) cited this part as 1904, and Zoological Record indicated 1904, which making this a confusing situation. We believe that 1903 is the correct publication year, because Aurivillius corrected it to 1903 in his 1923’s catalogue. Most authors cite the eighth part as year 1903 (Heffern 2005, but erroneously missed the first page 313; Makihara 1999; Heffern 2011 (corrected and added page 313)).										
1907	9.	AFZ	3(18): 1–39.	93–131	97–155	figs 35–41.	pl. 1: figs 1–9.	12 Sept. 1906	7 Feb. 1907	24 Sept. 1907
The correct publication date of the ninth part is surely 1907, and most of authors cited it correctly (Aurivillius 1912, 1922, 1923; Breuning 1944; Gressitt 1951; Podaný 1971; Nakamura et al. 1992; Hüdepohl and Heffern 2004; Heffern 2005, 2011; Makihara and Woro 2002; Morati and Bentanachs 2009; Juhel and Bentanachs 2011, 2012; Nakamura et al. 2014; Yokio and Heffern 2016; Bentanachs and Jiroux 2017; Bezark 2019; Lin and Yang 2019). However, some authors used the submission date of 1906 (e.g., Adlbauer 2002b); while some authors used the year 1908 for unknown reason (e.g. Makihara 1999).										
1908	10.	AFZ	4(17): 1–9.	133–141	156–167	figs 42–47	none	11 Mar. 1908	1 May 1908	30 Sept. 1908
1910	11.	AFZ	7(3): 1–44.	143–186	168–223	fig. 48	none	1 June 1910	24 Sept. 1910	25 Jan. 1911
The publication date of the 11^th^ part is confusing. The correct year should be 1910, since on the last page it was printed “Tryckt den 24 september 1910”, Aurivillius himself cited it as 1910 (Aurivillius 1912, 1922, 1923), and the Zoological Record also listed it as 1910. Most authors cited it correctly (Quentin 1956; Podaný 1968; Hüdepohl 1992; Martins 1997, 1998, 2002; Vives and Heffern 2001; Heffern 2002; 2005; Vives and Abang 2003; Monné 2005a, 2012, 2019a, d; Bousquet et al. 2009; Bentanachs et al. 2010; Gouverneur 2015; Monné and Monné 2015; Viktora 2015b; Bezark 2019; Lin and Yang 2019). However, some authors used 1911 because the whole seventh volume was published on January 25, 1911 (Napp 1993; Morati and Huet 2004; Löbl and Smetana 2010; Heffern 2011; Viktora and Tichý 2017).										
1911	12.	AFZ	7(19): 1–41.	187–227	224–291	figs 49–57	none	7 June 1911	8 Dec. 1911	23 Dec. 1911
1913	13.	AFZ	8(22): 1–35.	229–263	292–351	figs 58–68	none	4 June 1913	17 Oct. 1913	31 Dec. 1913
1914a	14.	AFZ	8(29): 1–54.	265–318	352–453	none	pl. 1: figs 1–9	25 Feb. 1914	9 May 1914	22 May 1914
Aurivillius’s own page numbers did not state numbers for separate plates, though when the text ended with odd numbers the even numbers could be considered as numbers for the separate plates. There was not page number for the separate plate of the 14^th^ part, since the text ended with an even number 54 (=318) and the 15^th^ part began with 1 (=319).										
1914b	15.	AFZ	9(8): 1–15.	319–333	454–483	none	none	9 Sept. 1914	4 Nov. 1914	15 May 1915
The publication date of the 15^th^ part is confusing. According to ICZN 21.2 and 21.8, the correct date should be 1914, since on the last page it was printed “Tryckt den 4 november 1914”, Aurivillius himself cited it as 1914 (Aurivillius 1922, 1923), and the Zoological Record also included it in 1914. Most authors cited it correctly (Adlbauer 2002; Juhel and Bentanachs 2011; Juhel 2014a).										
1916	16.	AFZ	10(19): 1–25.	335–359	484–523	figs 69–72	pl. 1: figs 1–9	7 June 1916	12 Aug. 1916	28 Nov. 1916
The page 26 (=360) is unnecessary to be included, though it doesn’t matter so much if it is included. On the page 26 (=360), there was no content written by Aurivillius, but only “Tryckt den 12 augusti 1916. / Uppsala 1916. Almqvist & Wiksells Boktryckeri-A.-B. / -360-” Also, there was an ending mark on the page 25 (=359). In a similar situation that happened to the 12^th^ part, 42 (= 228) normally was not included.										
1920	17.	AFZ	13(9): 1–43.	361–403	523–595	figs 73–81	none	10 Mar. 1920	6 Sept. 1920	11 Oct. 1920
The species number 523 was used twice. It was used for “523. *Hilarolea humeralis*” in the 16^th^ part, and again as “523. *Ophistomis splendida*” in the 17^th^ part. The publication date of this part is surely 1920 and few errors were made. Martins (2005) carelessly used 1922 but he corrected it to 1920 later (Martins 2011, 2014).										
1922	18.	AFZ	14(18): 1–32.	405–436	596–639	figs 82–112	none	7 Dec. 1921	4 April 1922	26 July 1922
The publishing year of the 18^th^ part is surely 1922. However, some authors used the submission date 1921 (e.g., Makihara, 1999), or even 1920 for unknown reasons (e.g. Makihara and Woro 2002).										
1923	19.	AFZ	15(25): 1–43.	437–479	640–711	figs 113–133	none	6 June 1923	31 Dec. 1923	Jan. 1924
The date of publication of the 19^th^ part is one of the most confusing cases, since different people used different years and for different reasons. The correct year should be 1923. Many authors used 1923 because of the “Tryckt den 31 december 1923”on the last page (Aurivillius 1928; Breuning 1939, 1944, 1950; Lane 1950; Gressitt and Rondon 1970; Hüdepohl 1985, 1988, 1992; Makihara 1999; Martins 1999, 2009, 2014; Lingafelter and Hoebeke 2002; Hüdepohl and Heffern 2004; Heffern 2005; Makihara and Woro 2002; Monné 2005a, b, 2012; 2019a, b, d; Monné and Napp 2005; Yokio and Niisato 2009; Vives 2011; Monné et al. 2016; Monné and Monné 2017; Bezark 2019). However, since Zoological Record cited 1924 for this work, so do the Titan database (Tavakilian and Chevillotte 2019). Less than half of authors used 1924 as the publication date (Lee 1987; Hüdepohl 1990; Heffern 2011; Lingafelter et al. 2014; Nakamura et al. 2014; Viktora and Tichý 2016; Vitali et al. 2017; Lin and Yang 2019). The first author of this paper Mei-Ying Lin was confused in this case (chose 1924 in the catalogue by Lin and Yang (2019) and an earlier version of this paper, but finally decided to choose the earlier date of “Tryckt den 31 december 1923”). For unknown reasons, 1925 was used by Löbl and Smetana (2010) in the Palaearctic catalogue, and this was followed by Viktora (2013), Viktora and Tichý (2017) and Lazarev and Murzin (2019). Makihara and Woro (2002) erroneously used 1922.										
1925a	20.	AFZ	17A(12): 1–21.	481–501	712–753	figs 134–140	none	4 June 1924	17 Feb. 1925	
The publication date of the 20^th^ part is 1925, however, some authors wrongly used the submission year 1924 from the first page (e.g., Makihara 1999; Makihara and Woro 2002).										
1925b	21.	AFZ	18A(9): 1–22.	503–524	754–795	figs 141–163	none	3 June 1925	17 Nov. 1925	1926
The publication date of the 21^st^ part is also one of the most confusing cases. We consider 1925 as the correct year for two reasons: a) “Tryckt den 17 november 1925” was printed on the last page, which is the correct year according to ICZN items 21.2 and 21.8; b) Titan database and the following literature used 1925 (Makihara 1999; Martins 1999; Heffern 2005; Monné 2005a, b, 2012, 2019a, b, d; Lin and Yang 2011; Lingafelter et al. 2014; Santos-Silva and Galileo 2016; Sudre et al. 2016; Bezark 2019; Lin and Yang 2019). However, both Zoological Record and the Palaearctic catalogue (Löbl and Smetana 2010) used 1926, as did the following (Gressitt 1940, 1951; Bousquet et al. 2009; Heffern 2011; Ślipiński and Escalona 2013; Lin 2017).										
1927a	22	AFZ	19A(17): 1–23.	525–547	796–844	figs 164–177	pl. 1: figs 1–6	1 June 1927	20 Sept. 1927	3 Nov. 1927
1927b	23	AFZ	19A(23): 1–41.	549–589	845–919	figs 178–202		14 Sept. 1927	21 Dec. 1927	25 Jan. 1928
The publication date of the 23rd part is also one of the most confusing cases. We consider 1927 as the correct year for two reasons: a) “Tryckt den 21 december 1927” was printed on the last page, which is the correct date according to ICZN items 21.2 and 21.8; b) Titan database also used 1927. The following literature used 1927 (Breuning 1944, 1950, 1956; Hüdepohl 1988; Heffern 2005; Weigel and Skale 2011; Vives 2012, 2015b, 2017; Lingafelter et al. 2014; Nakamura et al. 2014; Wallin et al. 2014; Rousset et al. 2016; Vitali 2016; Lin and Yang 2019). However, both Zoological Record and the Palaearctic catalogue (Löbl and Smetana 2010) used 1928, as did the following (Heffern 2011; Lazarev and Murzin 2019). “utkom 25. jan. 1928” was printed on the wrapper. We chose the earlier date based on ICZN item 21.8.										

b) Errors regarding part numbers. Sometimes authors cited the title without the part numbers (Hüdepohl 1985; Martins 1998; Nýlander 1998; Jiroux 2011; Juhel and Bentanachs 2011; Vives 2011; Monné et al. 2012; Juhel 2014a; Wallin et al. 2014; Ślipiński and Escalona 2016; Bezark 2019 for the second and third part), which is an incomplete citation. Sometimes authors made mistakes on the part numbers (for example, Hüdepohl (1992) wrote part 11 incorrectly as 2; Makihara (1999) wrote part 21 while the journal’s information indicates part 22; Vives (2009) wrote part 21–22 while the journal’s information indicate part 22, and he used the figure numbers as page numbers). Some authors mixed different parts together, such as Martins (2011) mixing part 13 and 15 together as “13. Arkiv Zool. 9(8): 229–263“, while the journal’s volume 9(8) belongs to part 15, not part 13.

c) Errors regarding first and last page numbers. Sometimes the first page number was missing (Heffern 2005, missing page 313 of part 8), or last page number was missing (Lingafelter and Hoebeke 2002, missing page 224 of part 7, and missing part 7 from the title too; Sudre et al. 2010, missing last 23 pages of part 12; Juhel and Bentanachs 2011, 2012, missing page 39 =131 of part 9; Juhel 2016, missing page 54 = 318 of part 14), or adding one more page (for example, Martins 1997, 1998, 2002, 2005; Monné 2005a, b, 2012; Monné and Monné 2015 and Bezark 2019, added 187 to part 11; Vives 2015d, Yan and Chen 2016 and Lazarev and Murzin 2019, added 228 to part 12). Adding 187 to part 11 is an error that should be corrected, because 187 is the first page number of part 12.

d) Errors regarding about journal volume numbers. Sometimes the volume numbers of the journal were wrongly cited. For example, Podaný (1971) wrote 3 (10) for part 9, while the correct number should be 3 (18); Bentanachs et al. (2010) wrote 7(2) for part 11, while the correct number should be 7 (3); Ohbayashi and Niisato (2007) wrote volume 21 for part 7, while the correct volume number should be 23.

e) Other errors. Some authors cited the figure numbers as page numbers (Vives 2009, 2011), or cited the part number as page number (Löbl and Smetana 2010; Lin 2015, 2017; see fig. 4), or cited page numbers erroneously for unknown reasons (Vives and Abang 2003).

**Figure 2.**
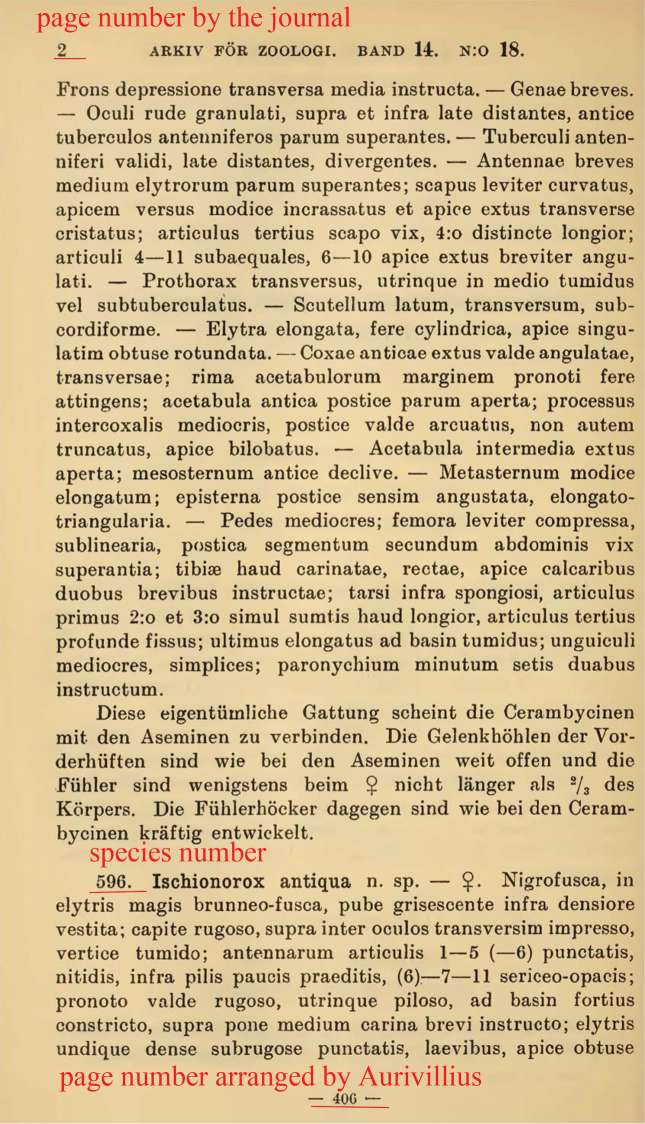
Second page of part 18, showing the species number, journal’s page number and Aurivillius’s page number.

**Figure 3.**
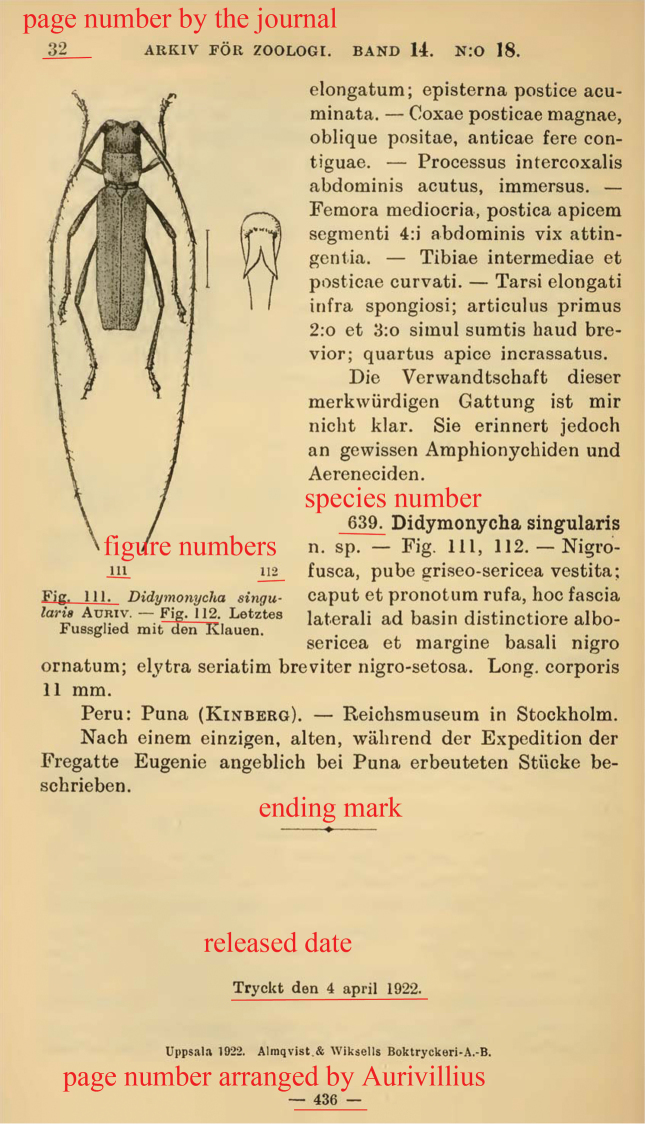
Last page of part 18, showing the species number, figure numbers, ending mark, release date, journal’s page number and Aurivillius’s page number.

**Figure 4.**
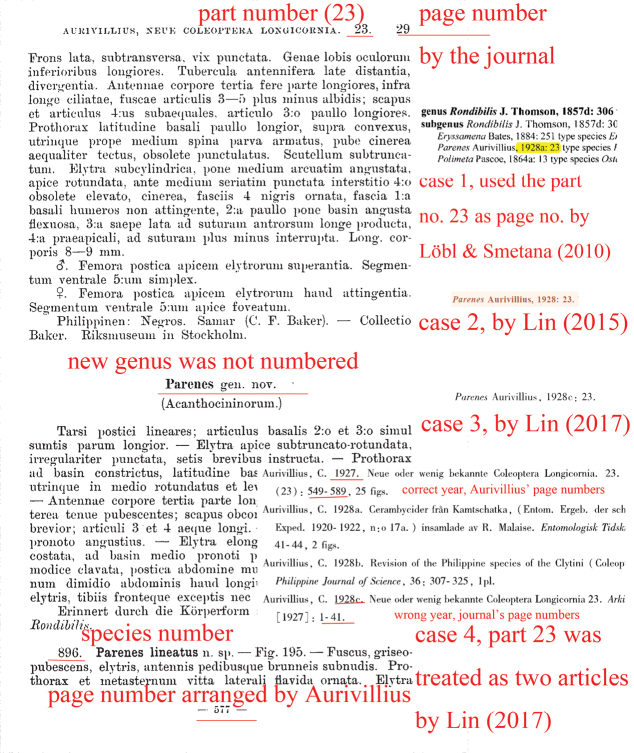
page 29 of part 23, showing the part number, species number, journal’s page number and Aurivillius’s page number, and four cases of wrong citation.

### Examples of other types of errors

Nakamura et al. (2014) and Lin (2017) cited part 23 twice in the same paper or book, they used the journal’s page numbers and Aurivillius’s own numbers in different places in the same publication, and used different years for the same part 23, which made part 23 look like two different articles. Hence, they made mistakes for citations of related taxa. Nakamura et al. (2014) used 1928 for the genus *Mimectatina* in the title, while the original article they used Aurivillius 1927: 27, then type species was written as *Mimectatina singularis* Aurivillius, 1928. Nakamura et al. (2014) variably used 1928 and 1927 in their authorship date for *Mimectatina*, causing confusion. And Nakamura et al. (2014) used the journal page number for the detailed taxon citation “*Cataphrodisium*: Aurivillius: 8”, while in the reference they used Aurivillius’s own page numbers “93–131”, which were incorrect. Nakamura et al. (1992) used 1927 for the type species *Mimectatina singularis* and 1928 for the genus *Mimectatina*. Lin (2017) used 1927 and Aurivillius’s page numbers for the genus *Mimectatina* (writing “*Mimectatina* Aurivillius, 1927: 575”, which should be corrected to “*Mimectatina* Aurivillius, 1927: 27 (= 575)”), then used 1928 and the journal’s page numbers for the genus *Parenes* (Fig. 4, writing “*Parenes* Aurivillius, 1928c: 23”, which was copied from Löbl and Smetana (2010) and should be corrected to “*Parenes* Aurivillius, 1927: 29 (= 577)”), wrongly treating the same paper as two separate articles.

## Discussion

### Date of publication we chose

The dates of publication of this series of work contain several confusing cases; the detailed information is shown in Table 1. For parts 5 and 6, we chose the earlier date indicated by the Zoological Record and Derksen and Scheiding (1963), instead of the later date printed low on the back side of the original wrapper, based on IZCN 21.8.1. For parts 11 and 15, we chose the earlier date printed on the last page and indicated by Zoological Record, instead of the later date indicated by the journal, also based on IZCN 21.8.1. For parts 19, 21 and 23, we chose the earlier date printed on the last page, instead of the later date indicated by Zoological Record and the journal, also based on IZCN 21.8.1, Before 2000, an author who distributed separates in advance of the specified date of publication of the work in which the material was published thereby advanced the date of publication.

When we talk about “distribute reprints in advance” in Aurivillius’s cases, the authors mean distribute the reprints after the printing date (“tryckt den XX YY 19ZZ”) but before the distribute date of the publisher (either printed on the wrapper, normally for the whole volume, or date applied subsequently by the Zoological Record).

### Why the journal page numbers should be used

For parts 8 to 23 of Aurivillius’s works, the reasons that the journal page numbers should be used include: 1) the works were first officially published in the journal; 2) the large book titled “Neue oder wenig bekannte ColeopteraLongicornia” does not exist; 3) Aurivillius himself used the journal page numbers instead of his own page numbers (Aurivillius 1912, 1922, 1923, 1928); 4) if Aurivillius’s own page numbers were chosen, the results are chaotic since the numbers continued between different journals, different years, and additionally, some parts were missing (Table 1: pages 1–14 and 77–92 were not printed); 5) if Aurivillius’s own page numbers were chosen, logically there should be pages preceeding them in the same volume. For example, considering “Arkiv för zoologi 13(9): 361–403” instead of “Arkiv för zoologi 13(9): 1–43”, logically there should exist “Arkiv för zoologi 13(9): 1–360” (or “Arkiv för zoologi 13: 1–360”), but this is not the case.

### How to identify which page number was the journal’s page number

1) the page number was printed on the upper left corner (of even pages) or the upper right corner (of odd pages), which was the style of the journal “Arkiv för zoologi” (Aurivillius 1917, 1919, 1925a, 1925b, 1926), except the first page normally appeared on the lower right corner; 2) each part of each volume was numbered from one, which was also the style of journal “Arkiv för zoologi” at that time (Aurivillius 1917, 1919, 1925a, 1925b, 1926).

### Aurivillius’s own numbers might be chosen for the following reasons

1) it was the choice of the Titan database (Tavakilian and Chevillotte 2019), which is the most exhaustive Cerambycidae database; 2) larger sized numbers appear more important (for some reasons), for parts 3 to 7, which also had two page numbers printed, all were cited with the correct journal’s page numbers, because they are larger than Aurivillius’s own page numbers (such as Wappes et al. 2011; Ślipiński and Escalona 2016; Souza 2016; Tavakilian and Chevillotte 2019); 3) page numbers on the mid-bottom are more noticeable than page numbers on upper left corner (of even pages) or upper right corner (of odd pages); 4) works were reprinted with the smaller page numbers even though they were originally from a book or journal with the larger page numbers; realizing this subsequent workers may have chosen the larger numbers; 5) to follow author’s citing Aurivillius’s own page numbers.

### The trend

**Figure 5.**
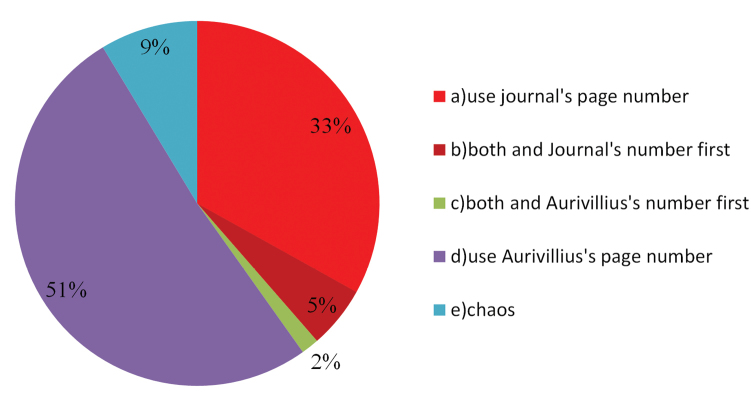
Ratios of different ways to cite Aurivillius’s series of works from 1912 to 2019.

**Figure 6.**
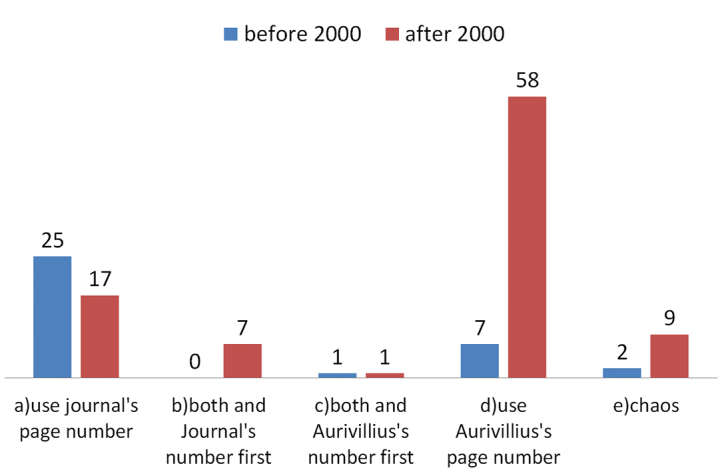
Ratios of different ways to cite Aurivillius’s series of works before and after the year 2000.

**Figure 7.**
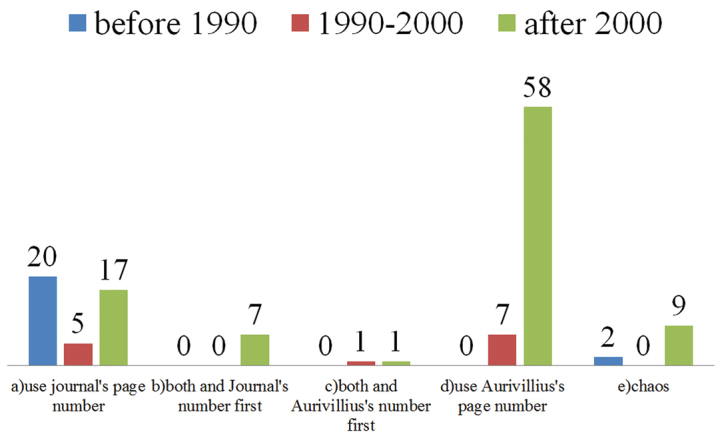
Ratios of different ways to cite Aurivillius’s series of works before the year 1990, between 1990–2000, and after the year 2000.

From Fig. 5 we can see that more than half of authors used Aurivillius’s page numbers instead of the journal’s page numbers. However, from Fig. 6 we can see that more authors used the journal’s page numbers than Aurivillius’s page numbers before the year 2000, while most authors used Aurivillius’s page numbers after the year 2000. Analyzing the references in more detail (Fig. 7), we can see that all authors before 1990 used the journal’s page numbers, while more and more authors used Aurivillius’s page numbers after 1991. The reasons for this trend might include: a) young authors did not know the history and might choose the bottom page numbers by the first glance; b) many current authors use the Titan database and copy the information from the website.

We hope that the Titan database will correct the information and use the journal’s page numbers after reading this paper, and authors in the future will cite the related references in correct way.

### Correct citation of Aurivillius’s works

Based on the above analyses, we suggest that in the future authors cite the work of Aurivillius as follows: the journal page number must be included, Aurivillius’s page numbers might be included inside square brackets [] or not included, the internal figure numbers (text-figures) can be included or not, while the supplemental information for the end-plates must be included.

Aurivillius, C. (1886) Nya ColeopteraLongicornia. Entomologisk Tidskrift 7(2): 89–94.

Aurivillius, C. (1887) Nya ColeopteraLongicornia. II. Entomologisk Tidskrift 8(4): 191–197. figs 1–3.

Aurivillius, C. (1891) Neue ColeopteraLongicornia. III. Entomologisk Tidskrift 12(2): 97–106 [=pp. 15–24], figs 1–6.

Aurivillius, C. (1893) Neue oder wenig bekannte ColeopteraLongicornia. 4. Entomologisk Tidskrift 14 (3): 177–186 [=pp. 25–34], figs 1–12.

Aurivillius, C. (1897) Neue oder wenig bekannte ColeopteraLongicornia. 5. Entomologisk Tidskrift 18 (4): 241–248 [=pp. 35–42], pl. 3: figs 1–8.

Aurivillius, C. (1899) Neue oder wenig bekannte ColeopteraLongicornia. 6. Entomologisk Tidskrift 20 (4): 259–265 [=pp. 51–57], figs 13–17.

Aurivillius, C. (1902) Neue oder wenig bekannte ColeopteraLongicornia. 7. Entomologisk Tidskrift 23: 207–224 [=pp. 59–76], figs 18–26.

Aurivillius, C. (1903) Neue oder wenig bekannte ColeopteraLongicornia. 8. Arkiv för zoologi 1: 313–328, figs 27–34.

Aurivillius, C. (1907) Neue oder wenig bekannte ColeopteraLongicornia. 9. Arkiv för zoologi 3(18): 1–39 [=pp. 93–131], pl. 1: figs 1–9; figs 35–41.

Aurivillius, C. (1908) Neue oder wenig bekannte ColeopteraLongicornia. 10. Arkiv för zoologi 4(17): 1–9 [=pp. 133–141], figs 42–47.

Aurivillius, C. (1910) Neue oder wenig bekannte ColeopteraLongicornia. 11. Arkiv för zoologi 7(3): 1–44 [=pp. 143–186], fig. 48.

Aurivillius, C. (1911) Neue oder wenig bekannte ColeopteraLongicornia. 12. Arkiv för zoologi 7(19): 1–41 [=pp. 187–227], figs 49–57.

Aurivillius, C. (1913) Neue oder wenig bekannte ColeopteraLongicornia. 13. Arkiv för zoologi 8(22): 1–35 [=pp 229–263], figs 58–68.

Aurivillius, C. (1914a) Neue oder wening bekannte ColeopteraLongicornia. 14. Arkiv för zoologi. Uppsala 8(29): 1–54 [=pp. 265–318], pl. 1: figs 1–9.

Aurivillius, C. (1914b) Neue oder wenig bekannte ColeopteraLongicornia. 15. Arkiv för zoologi 9(8): 1–15 [=pp. 319–333].

Aurivillius, C. (1916) Neue oder wenig bekannte ColeopteraLongicornia. 16. Arkiv för zoologi 10(19): 1–25 [=pp. 335–359], pl. 1: figs 1–9; figs 69–72.

Aurivillius, C. (1920) Neue oder wenig bekannte ColeopteraLongicornia. 17. Arkiv för zoologi 13(9): 1–43 [=pp. 361–403], figs 73–81.

Aurivillius, C. (1922) Neue oder wenig bekannte ColeopteraLongicornia. 18. Arkiv för zoologi 14(18): 1–32 [=pp. 405–436], figs 82–112.

Aurivillius, C. (1923) Neue oder wenig bekannte ColeopteraLongicornia. 19. Arkiv för zoologi 15(25): 1–43 [=pp. 437–479], figs 113–133.

Aurivillius, C. (1925a) Neue oder wenig bekannte ColeopteraLongicornia. 20. Arkiv för zoologi 17A(12):1–21 [=pp. 481–501], figs 134–140.

Aurivillius, C. (1925b) Neue oder wenig bekannte ColeopteraLongicornia. 21. Arkiv för zoologi 18A(9):1–22 [=pp. 503–524], figs 141–163.

Aurivillius, C. (1927a) Neue oder wenig bekannte ColeopteraLongicornia. 22. Arkiv för zoologi 19A(17): 1–23 [=pp. 525–547], pl. 1: figs 1–6; figs 164–177.

Aurivillius, C (1927b) Neue oder wenig bekannte ColeopteraLongicornia. 23. Arkiv för zoologi 19A(23): 1–41 [=pp. 549–589], figs 178–202.

## Supplementary materials

Supplementary material 1 Discussions and evidences Data type: personal communications / figures. https://binary.pensoft.net/file/380048This dataset is made available under the Open Database License (http://opendatacommons.org/licenses/odbl/1.0/). The Open Database License (ODbL) is a license agreement intended to allow users to freely share, modify, and use this Dataset while maintaining this same freedom for others, provided that the original source and author(s) are credited.Mei-Ying Lin, Si-Qin Ge
