# Supplementary material for: Aurivillius’s “Neue oder wenig bekannte ColeopteraLongicornia” (1886–1927), the correct years and page numbers
Source: Zookeys. 2020 Feb 12;911:113–37. doi: 10.3897/zookeys.911.48684 (PMC7031387; doi:10.3897/zookeys.911.48684)

**Discussions with colleagues by emails, parts attached here so that readers might have more informations.**

**2019-11-21**

Dear Mei-Ying,

This is an important contribution, good you decided to ake care of the case. Well, give me please some time, I will try to find out if the parts with separate page numbers issued also separately, and before the entire volume. If not, I think the volume pagination should be used as the more important one, already because when asking for a copy from libraries this provided unambiuous information.

I think best wozld be to ask for this information Michael Sörensson: **do you aree that I forward your mesage to him???**

By the way, "Index Litteraturae Entomologica, Serie 2" is the most important ntomological bibliography covering the years 1864-1900. It gives for the Aurivillius work part 5 the year **1897** ad for the part 6 the year **1899**.

As to the cerambycid sections of our Catalogue: together with Ales we had to take over missing information. Some authors missed synonyms, type species and parts of references.  So, as we have tried to complete all this and spend a lot of time on the work, we believed correct to be considered also as authors. Nevertheless, I cannot say who provided the Aurivillius references, and do not remember if I have re-checked any of them.

Looking forward your reply,

Best, Ivan

**2019-11-23**

Hi Meiying,

I've talked with Norm and we add this to our discussion:

We think it would be very hard to dispute that the dates of publication given by the actual journal are in any way inaccurate. Remember that, although we access the journal through BHL, it is **NOT** BHL that is providing the dates, those are pages from the actual journal that we provided links for.  The  "Index Litteraturae Entomologica, Serie 2"  is a secondary source, and we have no way of knowing where they got their data.   So we should use the primary source when it is available.

Cheers,

Steve

**2019-12-10**

Dear Mei-Ying!

Many thanks for recent e-mail and sorry for the delay in responding!

I think you made a very nice job regarding the Aurivillius works so far and I read your article with great pleasure. It is nice to see someone paying meticulous attention to these intricate problems of publication which are so important for nomenclatural reasons.

I have divided your request for help in two parts, the first concerning Aurivillius' early publications in ET (Entomologisk Tidskrift) [= ’Entomological Journal’, Swed.], the second concerning the subsequent publications in AfZ (Arkiv för Zoologi) (= ’Archive for Zoology’, Swed.]. In this first response I will present the results of my investigation of individual (separate) issues of ET during 1886-1902, as based on my private and complete copy/run of ET (1880-2019) preserved at home in Lund.

The Entomologiocal Society of Stockholm (Entomologiska Föreningen) was founded on the 14th of December 1880. Every year afterwards, it was commemorated by the final annual society meeting being held yearly on December 14th. Obviously, the society every following year aimed for having the last annual issue of ET printed and published by this date, although occasionally printing was delayed in some years. However, by studying the text and content of single issues, particularly the last of any year and the consecutive first of the up-coming year, it is possible to get reliable indications for rough dates of publication. In general, it seems that the society was able to keep the planned publication rate of four issues (March, June, September, December) pretty well, with a few exceptions. This implies that the dates of publication printed low on the original back wrapper of single issues are generally credible and could be used as official publication dates for nomenclatural purposes. Since no date of printing/publication was given for the first decade of volumes, one has to rely on indications as presented by society minutes and other related texts for these volumes.

(1) ET 7(2): 89-94 (1886): published after 12th of July 1886 by indication (p. 112).

(2) ET 8(4): 191-197 (1887): published in 'December' 1887 by indication. Inside page of front wrapper of issue 4 (last) states that publication of ET follows the scheduled plan and is signed 'Stockholm in December 1887'. Front wrapper also bears the year '1887' which indicates publication in December this year. Thus, 'December' is the correct publication date, or more exact: December 31st.

(3) ET 12(2): 97-106 (1891): "Utgifvet den 18 juni 1891" (= issued/published on June 18th 1891) (printed low on the back side of the original wrapper).

(4) ET 14(1-3): 177-186 (1893): "Utgifvet den 25 maj 1893" (= issued/published on May 25th 1893) (printed low on the back side of the original wrapper). -- Pages 177-186 belong in fact to volume 14(3) (see p. 177 subscript below) and should be cited accordingly, although issues 1-3 were  issued collectively and simultaneously, and bound together.

(5) ET 18(3-4): 241-248 (1897): "Utgifvet den 19 januari 1898" (= issued/published on January 19th 1898) (printed low on the back side of the original wrapper). -- Pages 241-248 belong in fact to volume 18(4) (see p. 241 subscript below) and should be cited accordingly, although issues 3-4 were  issued collectively and simultaneously, and bound together.

 (6) ET 20(4): 259-265 (1899): "Utgifvet den 23 januari 1900" (= issued/published on January 23rd 1900) (printed low on the back side of the original wrapper).

 (7) ET 23(2-3): 207-224 (1902): "Utgifvet den 20 september 1902" (= issued/published on September 20th 1902) (printed low on the back side of the original wrapper). -- Pages 207-224 belong in fact to volume 23(3) (see p. 209 subscript below) and should be cited accordingly, although issues 2-3 were  issued collectively and simultaneously, and bound together.

  As for the run of AfZ, I still await response from the Royal Swedish Academy of Sciences. I will let you know as soon as I get the information.

All the best,

Mikael in Lund

Mikael Sörensson

Lund University, Ecology Bldg.,

Sölvegatan 37, Inst. of Biology

SE-223 62 LUND

Sweden

21.8.1. Before 2000, an author who distributed separates in advance of the specified date of publication of the work in which the material was published thereby advanced the date of publication.

Dear Mei-Ying!

Many thanks for the last e-mail and please excuse my slow responding! I will respond to your questions as follows:

(1) Yes, you are free to put my notes and info in the supplementary material section.

(2) I recognize the meaning and content of the ICZN-paragraph 21.8.1. On the other hand, I realize that it is difficult to prove that the prints were printed and/or distributed in advance of the typed date of printing on the back side of the volumes/issues or the reprints. Therefore, I would certainly trust the date of publication (issue) or (for AfZ) the printing date ( = e.g. "tryckt den XX YY 19ZZ”), unless there is explicit demonstration of facts on the contrary.

(3) Until it has been proved otherwise (by library accession catalogues, or via private letters from Aurivillius himself), I do not think that Aurivillius distributed his reprints in advance (Mei-Ying Lin: when I talk distribute reprints in advance in Aurivillius’ cases, I means distribute the reprints after the printing date ("tryckt den XX YY 19ZZ”) but before the distribute date by the publisher (came out date sometimes printed on wrapper, normally for the whole volume)). This is mainly due to the fact that the date of printing is explicitly annotated and typed at the end of the text, low on the last page (see enclosed photos). I checked the few Aurivillius-reprints from AfZ present in my private entomological library at home, and it is immediately obvious that the volume year should not be used but rather the specifically typed date of printing at the text end. In addition, in case of the printing year being different from the volume year, the printing year usually is repeated in further places in the reprint, which indicates its potential credibility. In the enclosed photos of a reprint of the 20th part from volume 17A:12 (1924) the last printed page (p. 21/501) states ”Tryckt den 17 februari 1925” (= Printed on the 17th of February of 1925). In case the reprint had been printed/issued in advance, I find it hard to comprehend why a specific printing date should have been given. Why print information which is not true? Thus, I feel confident that the date of printing, as explicitly provided both in the reprint and in the actual volume of AfZ is perfectly credible and should be used for dating of the cerambycid nomenclature. The dates cited in the ’Web of Science’ provided by you are thus at least partly in error.

Below, you find the printing dates of the few, scattered reprints from the Arkiv för Zoologi-parts which I own in my private library. The exact date of printing (bold) is given low on the last page, and the printing year is repeated (at least) also on the back side of the last wrapper.

Band 1 (1903). – Neue oder wenig bekannte Coleoptera Longicornia. 8. Mitgeteilt am 14. Oktober 1903. – ”Tryckt den 27 november 1903” [= printed on the **27th of November 1903**].

Band 3:18 (1907). – Neue oder wenig bekannte Coleoptera Longicornia. 9. Mitgeteilt am 12. September 1906. – ”Tryckt den 7 februari 1907” [= printed on the **7th of February 1907**].

Band 4:17 (1908). – Neue oder wenig bekannte Coleoptera Longicornia. 10.  Vorgelegt am 11. März 1908. – ”Tryckt den 1 maj 1908” [= printed on the **1st of May 1908**].

Band 17A:12 (1924). – Neue oder wenig bekannte Coleoptera Longicornia. 20. Vorgelegt am 4 Juni 1924. – ”Tryckt den 17 februari 1925” [= printed on the **17th of February 1925**].

Band 18A:9 (1925). – Neue oder wenig bekannte Coleoptera Longicornia. 21. Vorgelegt am 3 Juni 1925. – ”Tryckt den 17 november 1925” [= printed on the **17th of November 1925**].

Band 19A:17 (1927). – Neue oder wenig bekannte Coleoptera Longicornia. 22. Vorgelegt am 1 Juni 1927. – ”Tryckt den 20 september 1927” [= printed on the **20th of September 1927**].

Band 19A:23 (1927). – Neue oder wenig bekannte Coleoptera Longicornia. 23. Vorgelegt am 14. September 1927. – ”Tryckt den 21 december 1927” [= printed on the **21st of December 1927**].

I hope this will be of some help and aid you further to make the proper and best decisions!

Best wishes,

Mikael in lund

**Cases with printing year being different from the volume year. We showed the final decision of this research at the beginning, and the figure from web of Science which means the date from the Zoological Record, and some reasons and arguments too.**

**Case 1**

Aurivillius, C. (1897) Neue oder wenig bekannte Coleoptera Longicornia. 5. *Entomologisk Tidskrift* 18 (4): 241–248 [=pp. 35–42], pl. 3: figs. 1–9. 1898


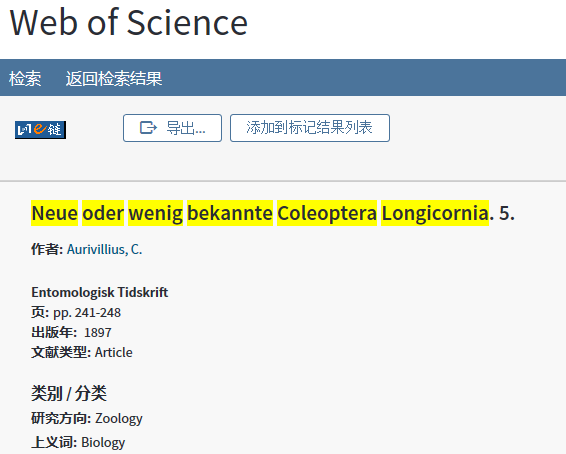


Mikael Sörensson: ET 18(3-4): 241-248 (1897): "Utgifvet den 19 januari 1898" (= issued/published on January 19th 1898) (printed low on the back side of the original wrapper). -- Pages 241-248 belong in fact to volume 18(4) (see p. 241 subscript below) and should be cited accordingly, although issues 3-4 were  issued collectively and simultaneously, and bound together.

Lin’s confusing: According to Derksen & Scheiding (1963), the date of publication is 1897. All references we searched and the Titan database (Tavakilian & Chevillotte 2019) used 1897, also the Zoological Record indicated 1897. However, the website <https://www.biodiversitylibrary.org/item/89782#page/8/mode/1up> indicated the publication date as 19 Jan. 1898. "Utgifvet den 19 januari 1898" (= issued/published on January 19th 1898) (printed low on the back side of the original wrapper) should be the official publication date. (personal communication with Mikael Sörensson on 10 December 2019).

**Case**

Aurivillius, C. (1899) Neue oder wenig bekannte Coleoptera Longicornia. 6. *Entomologisk Tidskrift* 20 (4): 259–265 [=pp. 51–57], figs. 13–17. 1900


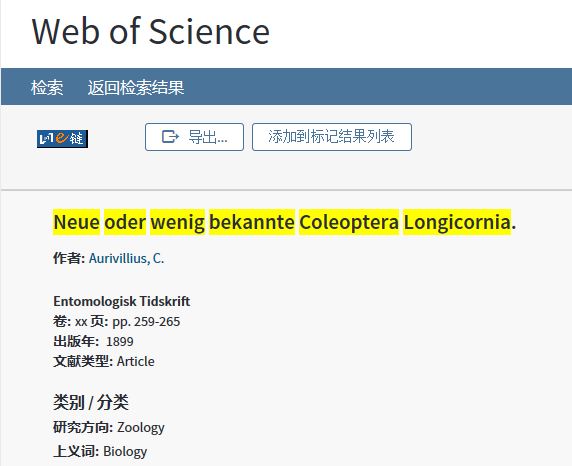


Mikael Sörensson: "Utgifvet den 23 januari 1900" (= issued/published on January 23rd 1900) (printed low on the back side of the original wrapper).

<https://www.biodiversitylibrary.org/item/43633#page/584/mode/1up>

Zoological Record indicates 1899.

**Case 3**

Aurivillius, C. (1903) Neue oder wenig bekannte Coleoptera Longicornia. 8. *Arkiv för zoologi* 1: 313–328, figs. 27–34. 1904


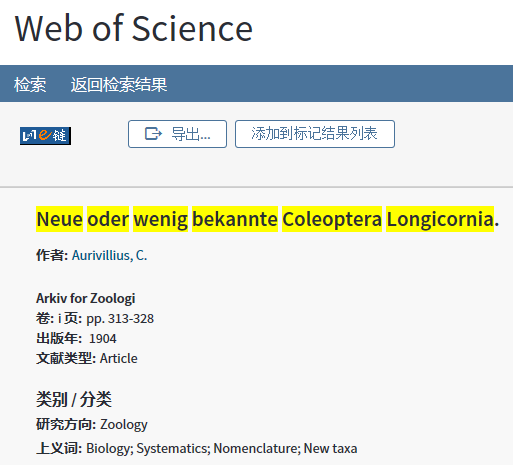


The date of publication of the eighth part is confusing. The printing date on the last page is “Tryckt den 27 november 1903”，so it should be 1903. However, Aurivillius (1912; 1922) cited this part as 1904, and Zoological Record indicated 1904, which making this a confusing situation. We believe that 1903 is the correct publishing year, because Aurivillius corrected it to 1903 in his 1923’s catalogue. Most authors cite the eighth part as year 1903 (Heffern 2005 (but erroneously miss the first page 313; Makihara 1999; Heffern 2011 (corrected and added page 313) ).

**Case 4**

Aurivillius, C. (1910) Neue oder wenig bekannte Coleoptera Longicornia. 11. *Arkiv för zoologi* 7(3): 1–44 [=pp. 143–186], fig. 48.


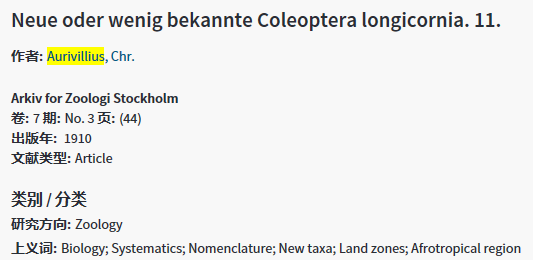


**Case 5**

Aurivillius, C. (1914b) Neue oder wenig bekannte Coleoptera Longicornia. 15. *Arkiv för zoologi* 9(8): 1–15 [=pp. 319–334].


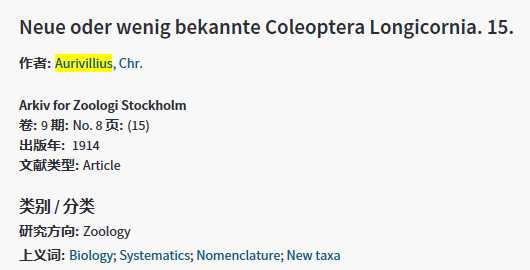


**Case 6**

Aurivillius, C. (1923) Neue oder wenig bekannte Coleoptera Longicornia. 19. *Arkiv för zoologi* [1923] 15(25): 1–43 [=pp. 437–479], figs. 113–133.


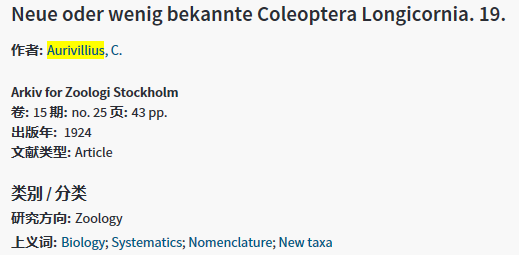


**Case 7**

Aurivillius, C. (1925b) Neue oder wenig bekannte Coleoptera Longicornia. 21. *Arkiv för zoologi* 18A(9):1–22 [=pp. 503–524], figs. 141–163.


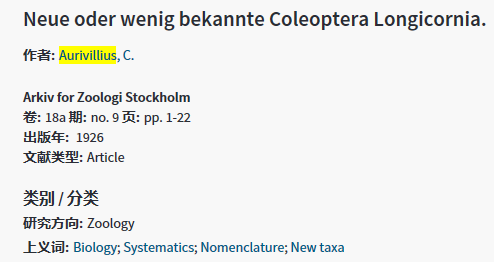


**Case 8**

Aurivillius, C (1927b) Neue oder wenig bekannte Coleoptera Longicornia. 23. *Arkiv för zoologi* 19A(23): 1–41 [=pp. 549–589], figs. 178–202.


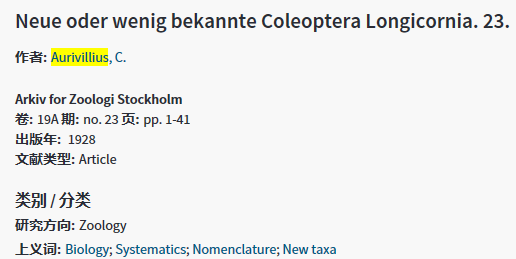

Supplement: Supplementary material 1 [file zookeys-911-113-s001.docx]
